# Limited Impact of Soil Microorganisms on the Endophytic Bacteria of Tartary Buckwheat (*Fagopyrum tataricum*)

**DOI:** 10.3390/microorganisms11082085

**Published:** 2023-08-15

**Authors:** Xuyan Liu, Xishen Zhu, Yumei Dong, Yan Chen, Meifang Li, Chengyun Li

**Affiliations:** 1 State Key Laboratory for Conservation and Utilization of Bio-Resources in Yunnan, Yunnan Agricultural University, Kunming 650201, China; xuyanliu@ynau.edu.cn (X.L.); zxs19960213@163.com (X.Z.); cyztz1001@163.com (Y.C.); lmf12290323@126.com (M.L.); 2 College of Plant Protection, Yunnan Agricultural University, Kunming 650201, China; 3 Yunnan-Taiwan Engineering Research Center for Characteristic Agriculture Industrialization of Yunnan Province, Yunnan Agricultural University, Kunming 650201, China; dymdym2006@126.com; 4 Yunnan-CABI Joint Laboratory for Integrated Prevention and Control of Transboundary Pests, Yunnan Agricultural University, Kunming 650201, China

**Keywords:** Tartary buckwheat (*Fagopyrum tataricum*), endophytic bacteria, sterile soil, bacteria diversity, co-occurrence network

## Abstract

Soil has been considered the main microbial reservoir for plants, but the robustness of the plant microbiome when the soil resource is removed has not been greatly considered. In the present study, we tested the robustness of the microbiota recruited by Tartary buckwheat (*Fagopyrum tataricum* Gaertn.), grown on sterile humus soil and irrigated with sterile water. Our results showed that the microbiomes of the leaf, stem, root and next-generation seeds were comparable between treated (grown in sterile soil) and control plants (grown in non-sterile soil), indicating that the plants had alternative robust ways to shape their microbiome. Seed microbiota contributed greatly to endophyte communities in the phyllosphere, rhizosphere and next-generation seeds. The microbiome originated from the seeds conferred clear benefits to seedling growth because seedling height and the number of leaves were significantly increased when grown in sterilized soil. The overall microbiome of the plant was affected very little by the removal of the soil microbial resource. The microbial co-occurrence network exhibited more interactions, and Proteobacteria was enriched in the root of Tartary buckwheat planted in sterilized soil. Our research broadens the understanding of the general principles governing microbiome assembly and is widely applicable to both microbiome modeling and sustainable agriculture.

## 1. Introduction

Tartary buckwheat (*Fagopyrum tataricum* Gaertn.) originated in the Himalayan mountainous areas of western China and is currently grown in Asian countries, including China, Japan and South Korea, as well as Canada and Europe [1]. Tartary buckwheat is a traditional short-season pseudocereal crop that performs well in barren soil and harsh climates. Compared with other crops, Tartary buckwheat shows high resistance to aluminum (Al) toxicity in acidic soils [2]. Tartary buckwheat has attracted increasing world-wide attention in recent years because it has a higher nutraceutical value compared with cereals; it is gluten-free, with a high total vitamin B content [3]; and it has much higher levels of antioxidants such as rutin [4,5,6]. These components have been associated with many potential health benefits such as reducing cholesterol levels, blood clots and high blood pressure [7,8].

Microorganisms are ubiquitous across the entire life cycle of plants, yet we are only just beginning to treat plant holobionts as indivisible entities [9]. Microbial communities play a vital role in host ecology and evolution by, for example, influencing fitness and growth [10], offering protection against herbivores or driving the evolution of multi-disease resistance [11]. The root growth of Arabidopsis is influenced by the interaction between a single bacterial genus (*Variovorax*) and other genera [12]. In return, for microbial colonization, the plant provides stable niches and photosynthetic products to the microbiota.

The plant holobiont comprises the plant and multiple fungal and bacterial species and is characterized by a dense network of multitrophic interactions, including both pathogens and mutualists [13]. The composition of plant microbiota can be influenced by the host genotype, its ecological niche, and biotic and abiotic factors. In nature, healthy plants will switch to being symptomatic for disease when the microbiota communities shift into dysbiosis. Seeds represent one of the most crucial stages of a plant’s life history. In natural and agricultural ecosystems, seeds serve not only to initiate the life cycle and reproduce but also to facilitate dispersal, to adapt and to persist in new environments [14]. Usually, seeds germinate, and the germinated seedlings not only face threats from various pathogens and herbivores in the soil but also respond to resource limitation and deficiencies in overall habitat suitability. These factors make the seed-to-seedling transition in both natural and agricultural systems one of the most serious challenges in a plant’s life cycle. Microorganisms, especially fungi in the soil, can adversely affect seed germination and seedling growth and development [15]. It might be inferred that seed germination or seedling survival would be enhanced by fungicide treatment compared to those of control seeds that do not receive the treatment. In *Sedum alfredii*, the transmission of endophytes from shoot cuttings to the rhizosphere was far more efficient in sterile soil than in normal soil, and the transmitted endophytes became a dominant component of the newly established root-associated microbiome. *S. alfredii* growth was significantly promoted when cultivated in sterile soil [16].

Although soil is an important reservoir of microorganisms, little work has been conducted to reveal holistically the source of microbiome composition across the numerous potential microbial niches represented by multiple plant organ and tissue types. Some studies have indicated that plant-associated bacteria can be recruited from the soil, while others have indicated that neither local site conditions nor host genotypes fully explain the assembly of plant microbiota [17]. Only when the endogenous seed microbiome is severely disrupted will the soil microbiome colonize the rhizosphere, and during the process of plant microbiome repair the seed microbiome takes priority over the soil microbiome [18]. The endophytic microbial community in the plant changes dynamically during growth and development and is affected by various abiotic and biotic factors such as soil conditions, biogeography, plant genotype, microbe–microbe interactions and plant–microbe interactions [19]. Despite these, bacterial seed endophytes are highly conserved in some plant species [20,21] and potentially provide the bulk of the species pool from which the seedling microbiome is recruited. The seed microbiome is the most promising candidate for plant microbiome engineering efforts.

Plants have evolved the ability to produce a vast array of specialized metabolites that aid adaptation to different environmental niches [22]. Microorganisms are one of the key environmental factors affecting plant growth, and growth promoters and biological control agents can influence the content of antioxidant activity in common buckwheat [23]. The arbuscular mycorrhizal colonization of Tartary buckwheat and the changes in the microbial community during Tartary buckwheat wine fermentation have been documented [24,25]. However, the influence of microorganisms that were already present in the soil before the plant was grown on plant fitness and development and plant microbiome assembly are unclear. Accurate knowledge of the structure, composition and function of Tartary buckwheat microbiota and their changes can help to identify beneficial microbiota that might assist the plant’s growth and development. In this study, we focused on elucidating the role of the seed microbiome on the plant microbial community’s diversity, structure, composition, function and abundance. We attempt to elucidate the potential sources of seed microbiota, leaf microbiota, stem microbiota and root microbiota with or without the influence of soil microbiota.

## 2. Materials and Methods

### 2.1. Soil Sterilization and Source of Tartary Buckwheat

The humus soil was fermented from plant residues, commonly used for raising flowers on family balconies, cultivating seedling for field crops. The humus soil used in this experiment was purchased from Anning Caopu Town Co., Ltd. (Kunming, Yunnan, China). The humus soil was packaged by the sterilization bag separately and placed in a damp heat sterilization pot under 121 °C and 0.1 mPa for 20 min. This was repeated two more times. Seeds of Tartary buckwheat were collected at Zhaotong Academy of Agricultural Sciences, Yunnan Province.

### 2.2. Sample Collection and Preparation

After removing the seed coat, seeds of Tartary buckwheat were disinfected in 1% sodium hypochlorite (NaClO) for 10 min and then rinsed with sterile water 5–6 times to remove any residue. For germination, the seeds were placed in a conical flask containing MS medium (Cat#M8521, Beijing Solarbio Science & Technology Co., Ltd., Beijing, China) without hormones. The seeds were incubated at 23 °C under 12 h/12 h light/dark for 3 days to facilitate germination. Then, we planted the germinated seeds in pots with sterilized or non-sterilized humus soil, with 5 seeds per pot. The pots were placed in the outdoor potted field of Yunnan Agricultural University. Irrigation with sterilized water, except some rainwater, but no more fertilizer was applied throughout the entire growth period until the seeds matured.

We assessed the microbial communities for five groups samples: (1) Tartary buckwheat parent seeds (FO); (2) roots (TR), stems (TS) and leaves (TL) planted on sterilized humus soil; (3) roots (CR), stems (CS) and leaves (CL) planted on non-sterilized humus soil; (4) seeds (TI) harvested from sterilized humus soil; (5) seeds (CI) harvested from non-sterilized humus soil. Seeds germinated on MS medium for three days were rinsed with sterile water, frozen in liquid nitrogen and stored at −80 °C for sampling of seed microbiota. In this study, we therefore define the “seed microbiota” as the seeding germinated on MS medium for three days since there was barely any microbial DNA obtained from seeds directly. The sampling of microbiota from roots, stems and leaves was as follows: roots were taken out of the pot completely. Large soil clumps were removed by shaking the root, and then fibrous roots were removed, and only the main roots were retained. Main roots were extensively washed by hand with tap water. Washed roots were transferred to sterile bags immersed in ice. Washed roots (2 g) were rinsed with sterile water 5–6 times, frozen in liquid nitrogen and stored at −80 °C. The tops of the stems were chopped and then washed by hand with tap water. Washed stems were transferred to sterile bags immersed in ice. The subsequent procedure was the same as for the roots. Young leaves were sampled following the same procedure as for stems.

### 2.3. DNA Extraction and Sequencing

All plant tissues (i.e., roots, stems, leaves and seeds) were ground to a powder in liquid nitrogen prior to DNA extraction. DNA extraction was performed using the MoBio PowerSoil DNA Isolation kit (QIAGEN, Hilden, Germany), according to the manufacturer’s protocol. DNA concentration and purity were monitored on 1% agarose gels. According to the concentration, DNA was diluted to 1 ng/µL using sterile water. To access the bacterial communities, the V4 region of the 16S rRNA gene was amplified using the specific primer (V4: 515F-806R) with the barcode. All PCR reactions were carried out in 30 µL reactions with 15 µL Phusion^®^ High-Fidelity PCR Master Mix (Biolabs, New England, Ipswich, MA, USA); 0.2 µM of forward and reverse primers, and about 10 ng of template DNA. PCR products were purified with the GeneJETTM Gel Extraction Kit (Thermo Scientific, Shanghai, China). Sequencing libraries were generated using Ion Plus Fragment Library Kit 48 rxns (Thermo Scientific, Shanghai, China) following the manufacturer’s recommendations. The library quality was assessed on the Qubit@ 2.0 Fluorometer (Thermo Scientific, Shanghai, China). The library was sequenced on an Ion S5TM XL platform (Novogene, Beijing, China), and 400 bp/600 bp single-end reads were generated.

### 2.4. Sequence Processing and Statistical Analysis

Single-end reads were assigned to samples based on their unique barcode and truncated by removing the barcode and primer sequence. Quality filtering of the raw reads was performed under specific filtering conditions to obtain high-quality clean reads according to the Cutadapt [26] quality control process. The reads were compared with the Silva138.1 using the UCHIME algorithm to detect chimera sequences, which were subsequently removed to give the final clean reads. Operational taxonomic units (OTUs) were then clustered at 97% similarity using Uparse [27] (v 7.0.1001). For each representative sequence, the Silva138.1 Database [28] was used with the Mothur algorithm to annotate taxonomic information [29]. Alpha diversity parameters, including observed species, Chao1, Shannon, Simpson and ACE, were calculated with QIIME [30] (v 1.9.1). Microbial community composition was assessed by computing Bray–Curtis dissimilarity matrices and then visualized using non-metric dimensional scaling (NMDS) ordinations to show compositional differences. We calculated unpaired *t*-test comparisons of bacterial communities within leaves, stems, roots and seeds using GraphPad Prism 8. Duncan’s new complex difference method in SAS v.9.0 (SAS Institute Inc., Cary, NC, USA) was used to conduct the analysis of variance (ANOVA), and the significant differences between different treatments in each experiment were compared at the level of *p* < 0.05.

### 2.5. Circos and Co-Occurrence Network

Graphical rendering of the community structure at phylum level was performed with the open-source software Circos [31]. From the abundance of the species, we calculated the correlation coefficient values (Pearson correlation coefficient, for each genus to obtain the correlation coefficient matrix with the filtering conditions set as follows: (a) cutoff value (>0.4) to filter out weakly related connections; (b) node self-joining filtered out; (c) connections with node sum abundance less than 8% removed. Network legends were created with Cytoscape. Topological features were estimated with igraph package in R.

## 3. Results

### 3.1. Comparison of Alpha Diversity in Different Tissues of Tartary Buckwheat

The experiment collected 135 samples for the amplicon sequencing of endophytic bacteria (Supplementary Table S1), and species accumulation showed sufficient sample size (Supplementary Figure S1). After the removal of low-quality reads and chimera sequences, we obtained on average 78,784 valid reads per sample. A total of 3364 bacterial operational taxonomy units (OTUs) were recovered, with clustering at ≥97% sequence identity using UPARSE. The taxonomic assignment of bacterial OTUs resulted in the identification of 66 phyla, including candidate phyla. Raw sequences of 16S DNA amplicons were submitted to NCBI as Bioproject PRJNA957132.

The microbial alpha diversity including the Simpson, ACE, Chao1 estimator and Shannon indices within each sample were analyzed based on the number of observed species for all sample types (Supplementary Table S2). Compared with the seedlings germinated for 3 days, alpha diversity indices in different tissues were slightly higher. And that in the leaf (TL) planted in sterile humus soil were significantly different from seedlings (FO) germinated for 3 days (Table 1, *p* < 0.05). But this trend was not found in the leaf (CL) planted in non-sterile soil. Whether planted in sterile soil or non-sterile soil, the microbial alpha diversity indices of the root endophytic bacteria were significantly different from those of the seedlings germinated for 3 days. This conclusion was consistent with the results of stem endophytic bacteria. But there was no significant difference between the same tissues planted in sterilized and non-sterilized soil.

The seedlings germinated for 3 days showed the lowest alpha diversity. Similar results were retrieved from the number of observed species, with the highest species numbers detected in the stem sample grown in sterile humus soil (Supplementary Table S2). The number of species observed for leaf endophytic bacteria was slightly less than in roots, but there were slightly more species detected in sterilized soil (TL) than in non-sterilized soil (CL) (observed species TL: 97, CL: 92). The number of observed species was the least in the 3-day-old seedlings. but that in parent seedlings (FO) was significantly less than in seedlings from non-sterilized soil (CI). The root endophytic bacteria community was also more diverse than aboveground stems and leaves regardless of whether planted in sterilized or non-sterile soil. 

### 3.2. Comparison of Plant Height and Leaf Number of Tartary Buckwheat Grown in Sterilized and Non-Sterilized Soil

**Figure 1:**
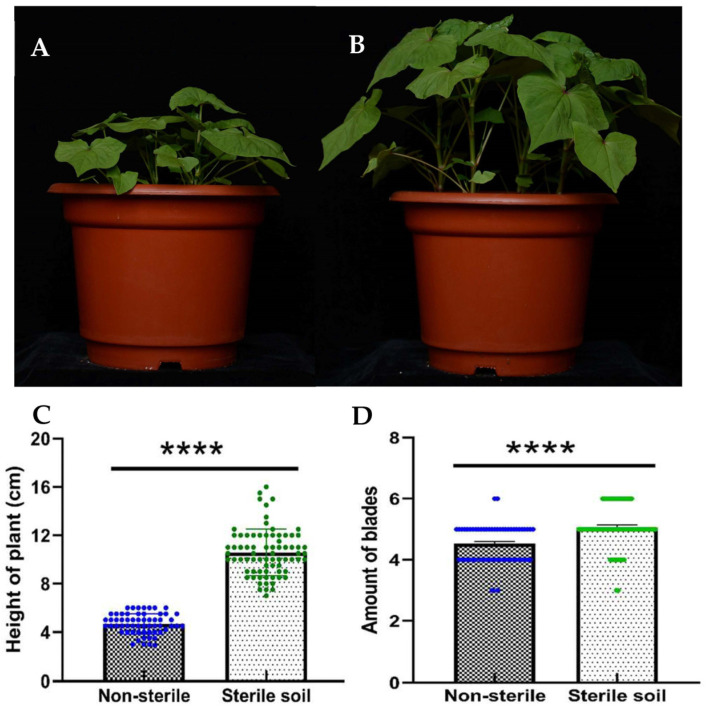
Comparison of the height and leaf number of Tartary buckwheat. Tartary buckwheat was planted in non-sterilized humus soil (**A**) and in sterile humus soil (**B**). Comparison of the height (**C**) and leaf number (**D**) of Tartary buckwheat planted in non-sterile or sterilized humus soil. **** indicates *p* < 0.0001.

Seedlings were transplanted into pots with sterilized or non-sterile humus soil. The height and leaf number of the plants were measured after 20 days. The average height of Tartary buckwheat grown in sterilized humus soil was 10.6 cm, with an average of 5.1 leaves per seedling, while the average height was only 4.7 cm, planted in non-sterilized soil. The height of Tartary buckwheat planted in sterilized soil was 2.25-fold greater than that in non-sterilized soil (Figure 1). The number of leaves was also significantly higher when grown in sterilized soil (*p* < 0.0001).

### 3.3. Assembly of Microbial Community in Different Plant Parts and Core Microbiome Identification

**Figure 2:**
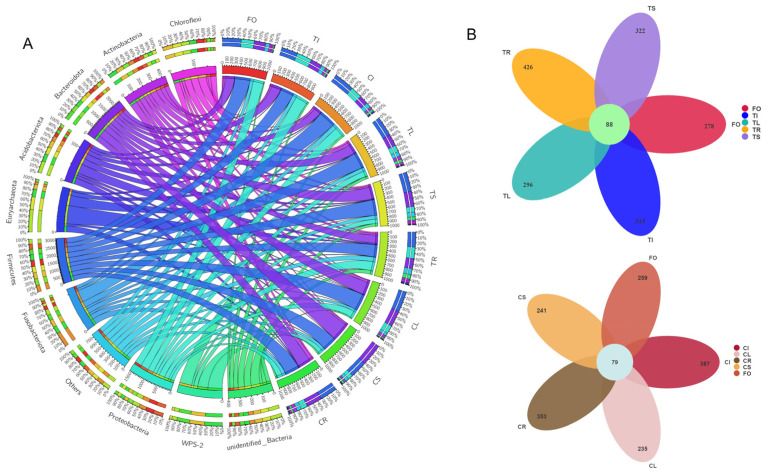
Composition of endophytic bacterial microbial community in different parts of Tartary buckwheat (**A**) and core microbiome (**B**). The seeds of Tartary buckwheat germinated on MS medium for 3 days (FO); the seeds harvested from Tartary buckwheat planted in sterilized (TI) and non-sterilized soil (CI); the leaf planted in sterilized (TL) and non-sterilized soil (CL); the stem planted in sterilized (TS) and non-sterilized soil (CS); the root planted in sterilized (TR) and non-sterilized soil (CR).

**Figure 3:**
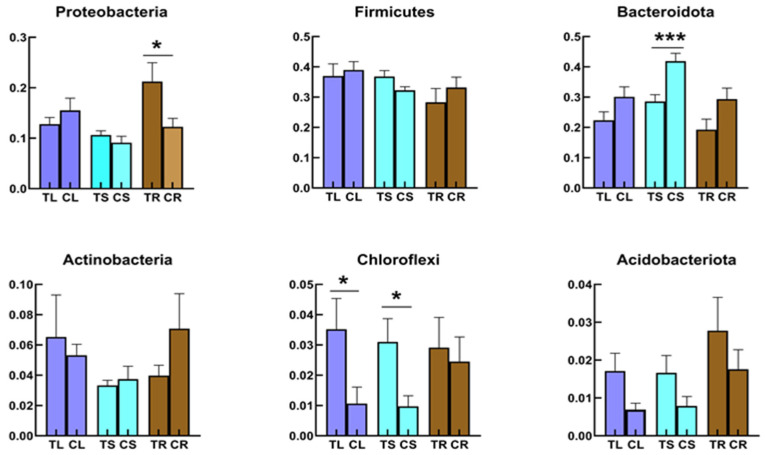
Comparison of relative abundance of bacterial community in phylum level in different tissues of Tartary buckwheat. The leaf planted in sterilized (TL) and non-sterilized soil (CL); the stem planted in sterilized (TS) and non-sterilized soil (CS); the root planted in sterilized (TR) and non-sterilized soil (CR). * indicates *p* < 0.05 and *** indicates *p* < 0.001.

At the phylum level of the bacterial community, all samples were dominated by Firmicutes (Figure 2A), which was present in most grain crops. In the stem of Tartary buckwheat grown in non-sterile humus soil, Bacteroidota were dominant (41%, Figure 2A), followed by Firmicutes and Proteobacteria (32% and 9%, respectively). The top three phyla in the root bacterial community from plants grown in non-sterile humus soil were Firmicutes (33%), Bacteroidota (29%) and Proteobacteria (12%). This was a little different from root samples grown in sterile humus soil. Although Firmicutes still had the highest relative abundance, Proteobacteria were the second highest in the root samples from sterilized soil and were significantly higher than those from non- sterilized soil (Figure 3; *p* < 0.05). The top three phyla in the leaf bacterial community grown in non-sterile humus soil were Firmicutes (39%), Bacteroidota (30%) and Proteobacteria (16%), followed by Actinobacteria (Figure 2A). There were no significant differences between plants grown in sterilized and non-sterilized soil (Figure 3; *p* < 0.05).

Proteobacteria were significantly enriched in the root samples planted in sterilized soil, compared with the bacterial community in leaves and stems (Figure 3; *p* < 0.05). In contrast, among the bacterial community in the root samples planted in non-sterilized soil, Proteobacteria were not enriched. The highest relative abundance of Proteobacteria occurred in the seedlings germinated for 3 days that were harvested from Tartary buckwheat planted in sterilized soil (Figure 2A). Proteobacteria were also enriched in seedlings germinated for 3 days that were planted in non-sterilized soil. Chloroflexi and Acidobacteriota were enriched more in roots, stems or leaves grown in sterilized soil than in non-sterilized soil (Figure 3). Bacteroidota were adverse. While Actinobacteria were more enriched in leaves grown in sterilized soil than in non-sterilized soil (Figure 3), it was just the opposite in root samples. Meanwhile, stem samples had higher relative abundances of Bacteroidota, compared with the leaves and roots samples planted in non-sterilized soil (Figure 3; *p* < 0.05).

**Figure 4:**
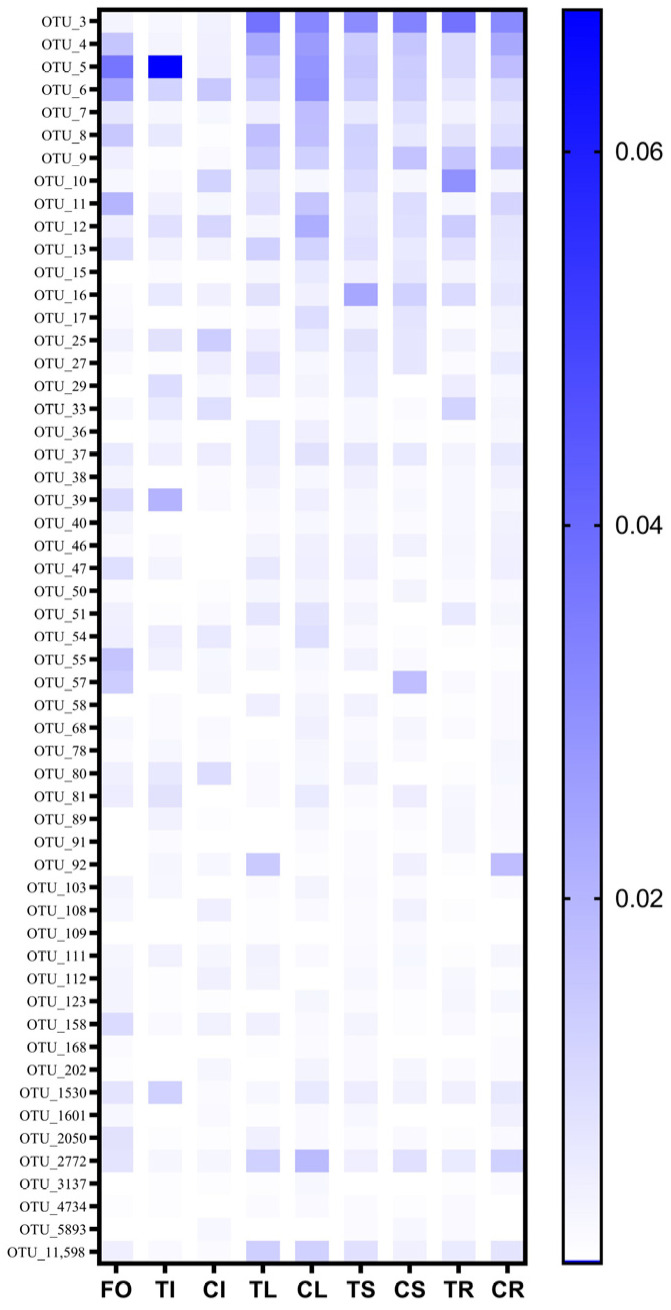
The relative abundance of core OTUs among different compartments of Tartary buckwheat. The seed of Tartary buckwheat germinated on MS medium for 3 days (FO); the seeds harvested from Tartary buckwheat planted in sterilized (TI) and non-sterilized soil (CI); the leaf planted in sterilized (TL) and non-sterilized soil (CL); the stem planted in sterilized (TS) and non-sterilized soil (CS); the root planted in sterilized (TR) and non-sterilized soil (CR).

Fifty-five prokaryotic OTUs were present in all samples at a minimum of 0.04% relative abundance and were identified as the core microbiome (Supplementary Figure S2). The 55 prokaryotic OTUs account for about 30% of the relative abundance in each group of samples. Firmicutes, Bacteroidota and Proteobacteria were among the most abundant members of the prokaryotic core microbiome. Core microbiome analysis showed that 79 and 88 prokaryotic OTUs were identified as core microorganisms grown on non-sterile soil and sterilized soil (Figure 2B), respectively. Soil sterilization has a significant impact on the endophytic core microorganisms of Tartary buckwheat. The relative abundance of 55 prokaryotic core OTUs in different compartments was also disturbed by soil sterilization (Figure 4). OTU_3, OTU_4 and OTU_5 were always at high relative abundance (>1%), regardless of tissue and soil type. OTU_7, OTU_11 and OTU_12 were only at high relative abundance (>1%) in leaves planted in non-sterilized soil. The relative abundance of OTU_92 in leaves planted in sterilized soil was 1.4%, but that was 0.1% in non-sterilized soil. Soil sterilization has little effect on the relative abundance of core endophytic bacteria in various tissues of Tartary buckwheat grown in sterilized and non-sterilized soil (Supplementary Figure S3).

**Figure 5:**
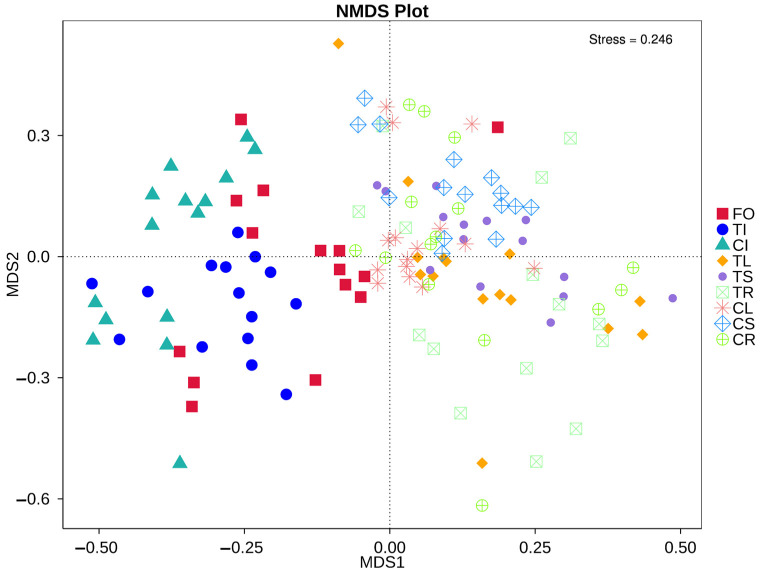
NMDS ordination of Bray–Curtis dissimilarity matrix obtained with bacterial communities in different tissues of Tartary buckwheat. The seed of Tartary buckwheat germinated on MS medium for 3 days (FO); the seeds harvested from Tartary buckwheat planted in sterilized (TI) and non-sterilized soil (CI); the leaf planted in sterilized (TL) and non-sterilized soil (CL); the stem planted in sterilized (TS) and non-sterilized soil (CS); the root planted in sterilized (TR) and non-sterilized soil (CR).

Plant tissue (root, stem, leaf and seedling) was found to be the major explanatory variable of microbial community structure NMDS (stress = 0.246; Figure 5). The structures of the seedlings and the different tissues of microbial communities were distinct. Although soil sterilization affected the relative abundance of the microbial community in the roots, stems and leaves, it did not affect the microbial community retained in the seeds and the community structure in different tissue.

### 3.4. Co-Occurrence Network of the Bacterial Community in Different Tissues of Tartary Buckwheat

**Figure 6:**
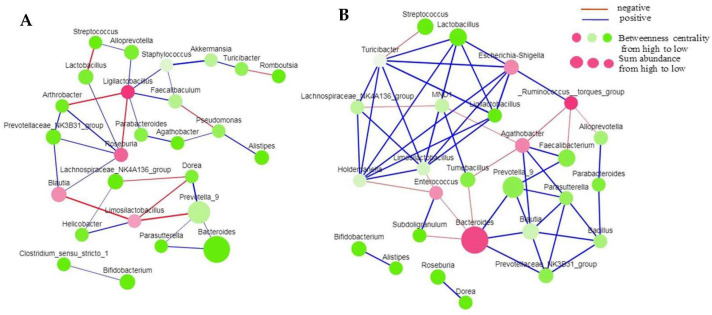
Co-occurrence network of bacteria in stem of Tartary buckwheat planted in non-sterilized soil (**A**) and in sterilized soil (**B**).

To examine variations in the microbial network structure, we built co-occurrence networks of the bacterial community in different tissues of Tartary buckwheat planted in non-sterile and sterilized soil. To construct the network, we selected approximately 30 genera, each with a sum relative abundance of over 7% in fifteen biological duplicate samples. The differences in the response of the bacterial communities detected in the different tissues suggest that the overall co-occurrence patterns of genera in the non-sterilized and sterilized soil would be different from each other. The co-occurrence network in stems from non-sterile soil consisted of 26 nodes and 33 edges, whereas that from sterilized soil consisted of 27 nodes and 52 edges (Figure 6). In roots from non-sterile soil, the co-occurrence network consisted of 26 nodes and 32 edges (Supplementary Figure S4A), whereas that from sterilized soil consisted of 27 nodes and 45 edges (Supplementary Figure S4B). In leaves from non-sterile soil, the co-occurrence network consisted of 26 nodes and 45 edges (Supplementary Figure S5A), whereas that from sterilized soil consisted of 29 nodes and 61 edges (Supplementary Figure S5B). The nodes and edges of the co-occurrence network suggested tighter associations among genera in sterilized soil than in non-sterile soil. Among the bacterial–bacterial networks, we recorded the high range proportion of positive edges in all tissues of Tartary buckwheat planted in sterilized or non-sterile soil.

### 3.5. The Proportion of Endophytic Bacteria in Different Tissues of Tartary Buckwheat 

**Figure 7:**
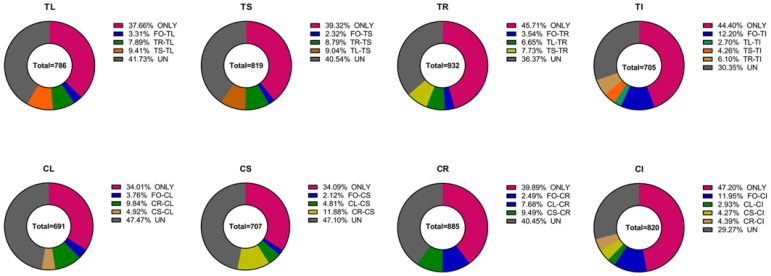
The proportion of endophytic bacteria in different tissues of Tartary buckwheat. ONLY: OTUs that only appeared in this diagram. FO-TL: OTUs that appeared simultaneously in FO and TL and so on. UN: OTUs that appeared in more than three tissues simultaneously. The seeds harvested from Tartary buckwheat planted in sterilized (TI) and non-sterilized soil (CI); the leaves planted in sterilized (TL) and non-sterilized soil (CL); the stems planted in sterilized (TS) and non-sterilized soil (CS); the roots planted in sterilized (TR) and non-sterilized soil (CR).

The Venn analyses of endophytic bacteria in different tissues of Tartary buckwheat in sterilized and non-sterilized soil (Supplementary Figure S6) show that only 45.7% of the endophytic bacteria in the roots (TR) grown in sterilized soil appeared (Figure 7). The proportion of root endophytic bacteria shared only with leaves (TL) grown in sterilized soil was 6.7%. And the proportion of root endophytic bacteria shared only with stems (TS) grown in sterilized soil was 7.7%. But this does not necessarily indicate the source of these endophytic bacteria. Furthermore, 36.4% of endophytic bacteria in the roots was shared with the stems, leaves, or parent seedlings simultaneously. But the proportion of recruited self-endophytic bacteria in Tartary buckwheat roots (TR), stems (TS) and leaves (TL) planted in sterilized soil was higher than that planted in non-sterile soil. The proportion of endophytic bacteria recruited by the offspring seedling (CI) from non-sterile soil was significantly higher than that in sterilized soil.

## 4. Discussion

Tartary Buckwheat is a traditional short-season pseudocereal crop that is adapted to growth in barren soil. Bacterial microbiota could promote nutrient uptake and transport from the soil as well as increase host immunity, increase tolerance to biotic (and abiotic stresses), promote stress resistance, and influence crop yield and quality. Similarly to human gut microbiota, the plant microbiome is referred to as the host’s second or extended genome. Soil was once considered the main source of plant microbiome, but more evidence suggests that the microorganisms in seeds are a valuable asset left by plants to their descendants. Plant–microbe interactions are of specific interest, not only to achieve a better understanding of their role during plant growth and development [32] but also to allow the exploitation of their relationships in phytoremediation applications [33], sustainable crop production [34], and the production of secondary metabolites [35].

### 4.1. Relationship between Soil Microbiota and Tartary Buckwheat Microbiome

In this study, we adopted a standard moisture–heat procedure, using hydrothermal by autoclave at 121 °C at 0.1 mPa for 20 min, three times, to reduce the influence of microorganisms that existed in soil on Tartary buckwheat growth. To investigate the function of rhizosphere microbiota, previous studies used a chemical method, involving the use of methyl bromide, chloropicrin or vancomycin [36], which kill or inhibit cell wall biosynthesis in certain taxa [37]. Plants are rooted and fixed in the soil and greatly rely on their root microbiome for the uptake of nutrients and protection against stresses. The life processes in which plants can survive under adverse environmental conditions are the result of co-evolution of plants and beneficial microorganisms [38]. For example, plants can in effect “cry for help” from their root microbiome when they are under attack by pathogens, leading to the selective enrichment of plant-protective microbes and microbial activities in the soil. Soil is considered to be a major reservoir of leaf [39] and root microorganisms for many plants and the main source of beneficial microorganisms for plants. Some studies on the origin of plant microorganisms have indicated that the phyllosphere largely comprises microbes that are passively dispersed and stochastically assembled from the atmosphere [40,41], but other studies in sugarcane [42,43], grape [44] and Arabidopsis [45] have suggested soil origins of leaf microbiota. Improved knowledge of the source of Tartary buckwheat microbiome is expected to aid our understanding of the mechanisms used to adapt to barren areas, and the identification of beneficial microorganisms, to improve the applicability of crops to different habitats. While our data suggested that 30% of bacteria observed in the roots, stems, and leaves could be detected in seedlings that had germinated for 3 days on an MS medium. The relative abundance of those bacterial OTUs could reach more than 50% in tissue. Therefore, the source of Tartary buckwheat microbiome might be attributed to non-soil reservoirs, with the vast majority derived from seed.

It is noteworthy that, according to the protocol of the soil microbial extraction kit, it is difficult to extract microbial DNA from the humus soil we used to grow the plants. Therefore, we believe that humus soil contains fewer microorganisms than natural field soils equal in quality. The plant height and leaf number of Tartary buckwheat grown in sterilized soil were significantly higher than in non-sterilized soil, and the endophytic bacteria of roots grown in sterilized soil was significantly more abundant than those of roots grown in non-sterilized soil.

### 4.2. Seed Microbiota Associated with Endophytic Microorganisms of Tartary Buckwheat

Seeds represent an essential stage of the plant life cycle and can be dormant for decades, under appropriate conditions, before germinating to produce new plants. Seeds contain complex microbial communities, which can exert beneficial effects on germination and support subsequent plant growth. Germination and emergence also can shape the structure of seed microbiota [46]. Although the microbial community might have been in dynamic change during plant growth and development, the endophytic bacterial communities are relatively well preserved and are transmitted to the next generation. This was confirmed in this study by the result that the microbial community structure of Tartary buckwheat seeds germinated for three days was very similar to that of the parent plants. The vertical transmission of microorganisms from seeds to seedlings has been studied in many crops, such as rice [47] and tomato [48], by culture-dependent experiments or 16S rRNA gene pyrosequencing. Previous studies showed that seeds might provide an important source of microbial inoculum for other plant organs, such as roots, stems, and leaves [49]. In this study, the data led us to assist this hypothesis. These microorganisms are well preserved in the seeds or seedlings and can be transferred to various plant tissues where they have the potential for plant growth promotion and the biocontrol of pathogens. Seed microorganisms can prevent the transmission and colonization of pathogens by maintaining tight hub networks and have a key role as carriers of plant-growth-promoting bacteria (PGPB). These interactions of the microbiome can be explained as an adaptation to prevalent environmental conditions as a result of the co-evolution of plants and microorganisms. In general, the higher the diversity of the microbial community, the greater the plant biomass that can be obtained. This is possibly because of not only effects on resource allocation but also enhanced evolution, allowing the microbiome to develop new desirable functions. The present study provides novel insights into bacterial endophytes of Tartary buckwheat.

In the last decade, rapidly increasing numbers of studies have focused on the role of the microbial community in various crops [50,51,52,53] Microbial abundance, composition, and function have a direct causal relationship with plant health [54]. Plant disease occurrence is often accompanied by microflora dysbiosis, such as altered microbial abundance or diversity [55]. Soil sterilization can change the relative abundance of specific genera in Tartary buckwheat microorganisms and the diversity of communities in different tissues.

In addition to microbial community structure and diversity, microbial co-occurrence patterns play a crucial role in understanding the plant microbiome. Previous studies have shown that the microbial co-occurrence network was modulated by environmental factors such as water depth [56] or soil properties [57]. Moreover, variations in microbial co-occurrence networks might represent different niches [58], and the presence of particular microbial modules might also indicate a similarity of microbial co-occurrence patterns in different environments. Identifying the hubs in a co-occurrence network can be used to infer their potential role in the community. Here, we reported that three tissues (leaf, stem and root) of Tartary buckwheat showed different co-occurrence patterns, which might also be influenced by the type of soil.

In our work, the numbers of nodes and edges of the co-occurrence networks in the stem of Tartary buckwheat grown in sterilized humus soil were significantly higher than those in non-sterilized soil. The most abundant genus did not display the highest number of interactions. According to the degree of betweenness centrality, the top three genera in the co-occurrence network in the stem of plants grown in sterilized humus soil were *Ruminococcus*, *Bacteroides* and *Agathobacter*. In non-sterile humus soil, the top three genera were *Ligilactobacillus*, *Roseburia* and *Blautia*. The same microorganisms might form different interactions in different niches, and the functional role of microorganisms is related to their niche. Therefore, this reminds us that, to fully understand the plant microbiome, its niche and environmental factors must be taken into account.

## 5. Conclusions

Like other cereal crops, Firmicutes were the dominant flora of Tartary buckwheat and were vertically transmitted to the next generation. Plant growth was significantly promoted when cultivated in sterile soil. The relative abundance of Chloroflexi, Bacteroidetes, and Firmicutes species in the leaves and roots were significantly affected by whether the soil was sterilized. The assembly of the microbiome in plant tissues was influenced by soil sterilization and interactions in the microbial community.

Overall, our findings suggest that non-soil microbes, especially seed-borne microorganisms, play a key role in plant growth and development. We believe that microorganisms in seeds are more conducive to plant growth and development. Microorganisms in soil have a weak impact on the distribution of seed microbiota in plant tissues. Detailed mechanisms on microorganisms in soil influence the colonization of seed endophytes in different plant tissues, and the scope of methods that could usefully enhance that colonization remains to be explored.

## Figures and Tables

**Table 1:** Alpha indices from Kruskal–Wallis analysis of bacterial community between seedings (FO) and different tissues of Tartary buckwheat.

Groups	Simpson		Shannon		chao1		ACE	
	*p*-Value	Sig	*p*-Value	Sig	*p*-Value	Sig	*p*-Value	Sig
FO vs. TL	0.027	*	0.014	*	0.023	*	0.018	*
FO vs. TS	3.00 × 10^−4^	***	1.00 × 10^−4^	***	0.084		0.151	
FO vs. TR	0.023	*	0.003	**	9.00 × 10^−4^	***	0.004	**
FO vs. CL	0.566		0.111		0.531		0.322	
FO vs. CS	0.006	**	0.005	**	0.904		0.968	
FO vs. CR	0.003	**	0.001	**	0.023	*	0.012	*
CL vs. TL	0.414		0.371		0.098		0.165	
CS vs. TS	0.386		0.273		0.065		0.140	
CR vs. TR	0.477		0.731		0.278		0.681	

Note: ***, ** and * indicate *p* < 0.001, *p* < 0.01 and *p* < 0.05, respectively.

## Data Availability

The datasets presented in this study can be found in online repositories. Raw sequence of 16S DNA amplicons were submited to NCBI as Bioproject PRJNA957132.

## Supplementary Materials

The following supporting information can be downloaded at: https://www.mdpi.com/article/10.3390/microorganisms11082085/s1. Figure S1: The endophytic bacteria species accumulation boxplot all types of sample; Figure S2: The core microbiome in different parts of Tartary buckwheat; Figure S3:The relative abundance of core OTU of Tartary Buckwheat grown in sterilized (A) and non-sterilized soil (B); Figure S4: Co-occurrence network of bacteria in root of Tartary buckwheat planted in non-sterilized (A) and sterilized soil (B); Figure S5: Co-occurrence network of bacteria in leaf of Tartary buckwheat planted in non-sterilized (A) and sterilized soil (B); Figure S6: Venn analysis of endophytic bacteria in different tissues of Tartary buckwheat in sterilized (A) and non-sterile soil (B); Table S1: Characteristics of effective reads and OTUs from samples of endophytic bacteria with Tartary buckwheat; Table S2: The Alpha diversity of bacterial community in seeding and different tissues of Tartary buckwheat.
