# Supplementary material for: Limited Impact of Soil Microorganisms on the Endophytic Bacteria of Tartary Buckwheat (Fagopyrum tataricum)
Source: Microorganisms. 2023 Aug 15;11(8):2085. doi: 10.3390/microorganisms11082085 (PMC10458046; doi:10.3390/microorganisms11082085)
Supplement: Supplementary file 1 [file microorganisms-11-02085-s001.zip › microorganisms-2522291-SI.pdf]

Supplementary Material

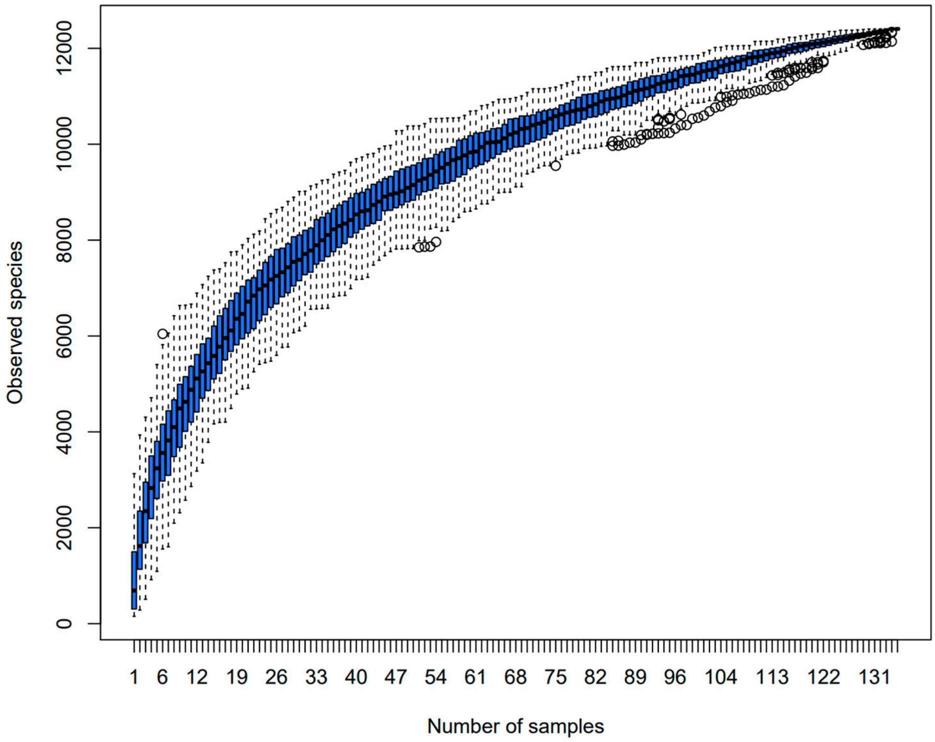

Figure S1. The endophytic bacteria species accumulation boxplot all types of sample.

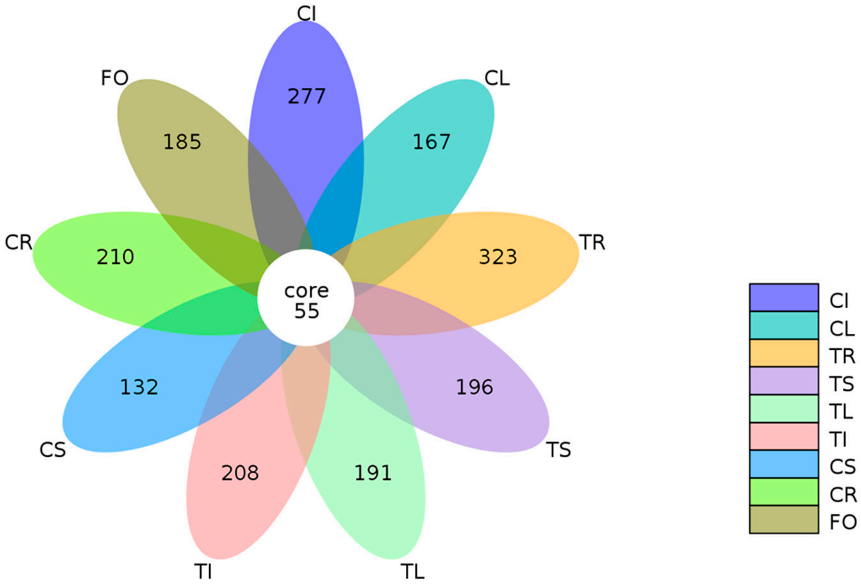

Figure S2. The core microbiome in different parts of Tartary buckwheat. FO: The seeds of Tartary buckwheat germinated on MS medium for 3 days; The seeds harvested from Tartary buckwheat planted in sterilized (TI) and non-sterilized soil (CI); The leaf planted in sterilized (TL) and non-sterilized soil (CL); The stem planted in sterilized (TS) and non-sterilized soil (CS); The root planted in sterilized (TR) and non-sterilized soil (CR).

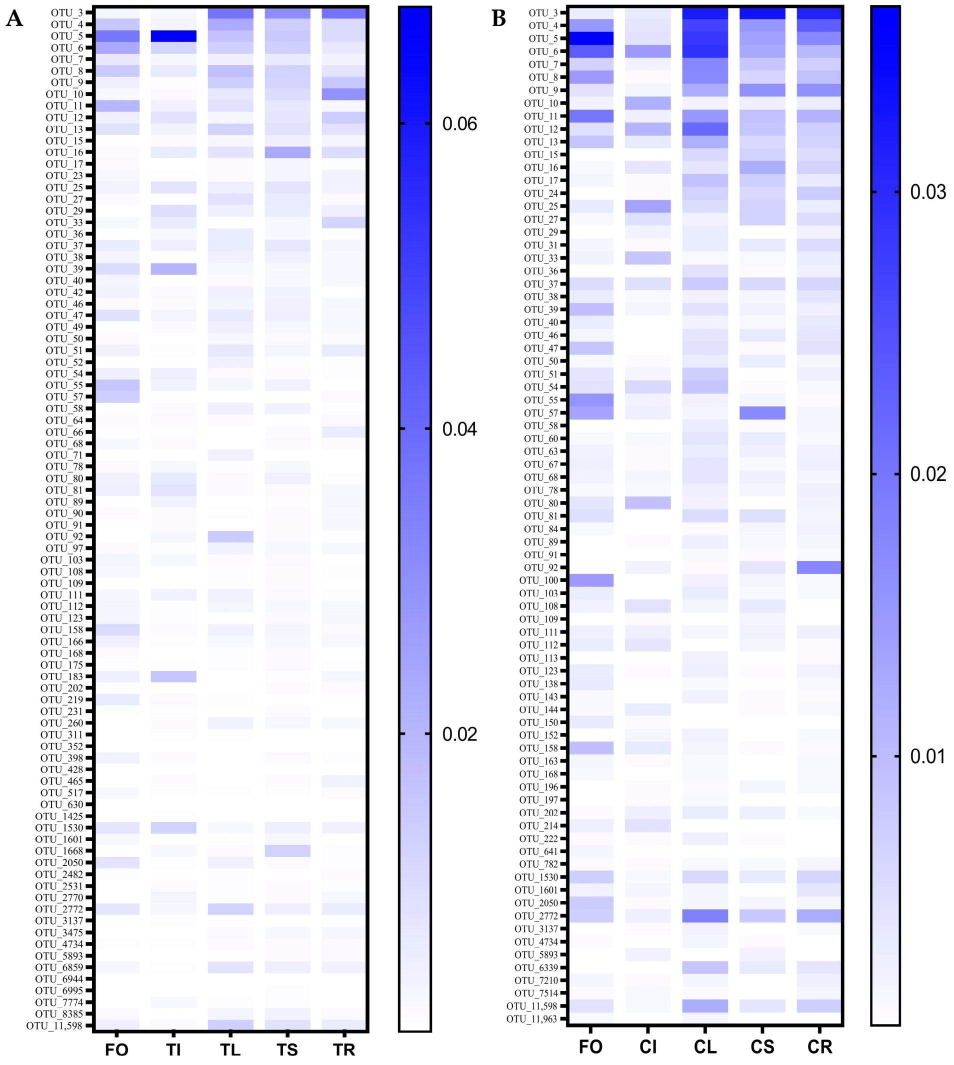

Figure S3. The relative abundance of core OTU of Tartary Buckwheat grown in sterilized (A) and non-sterilized soil (B). FO: The seeds of Tartary buckwheat germinated on MS medium for 3 days; The seeds harvested from Tartary buckwheat planted in sterilized (TI) and non-sterilized soil (CI); The leaf planted in sterilized (TL) and non-sterilized soil (CL); The stem planted in sterilized (TS) and non-sterilized soil (CS); The root planted in sterilized (TR) and non-sterilized soil (CR).

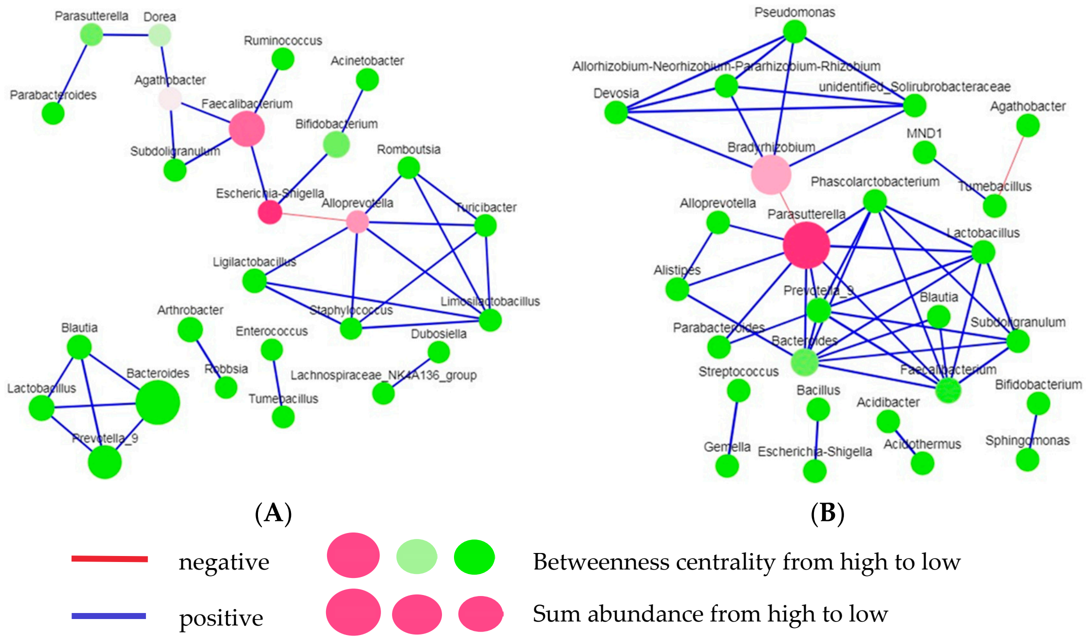

Figure S4. Co-occurrence network of bacteria in root of Tartary buckwheat planted in non-sterilized (**A**) and sterilized soil (**B**).

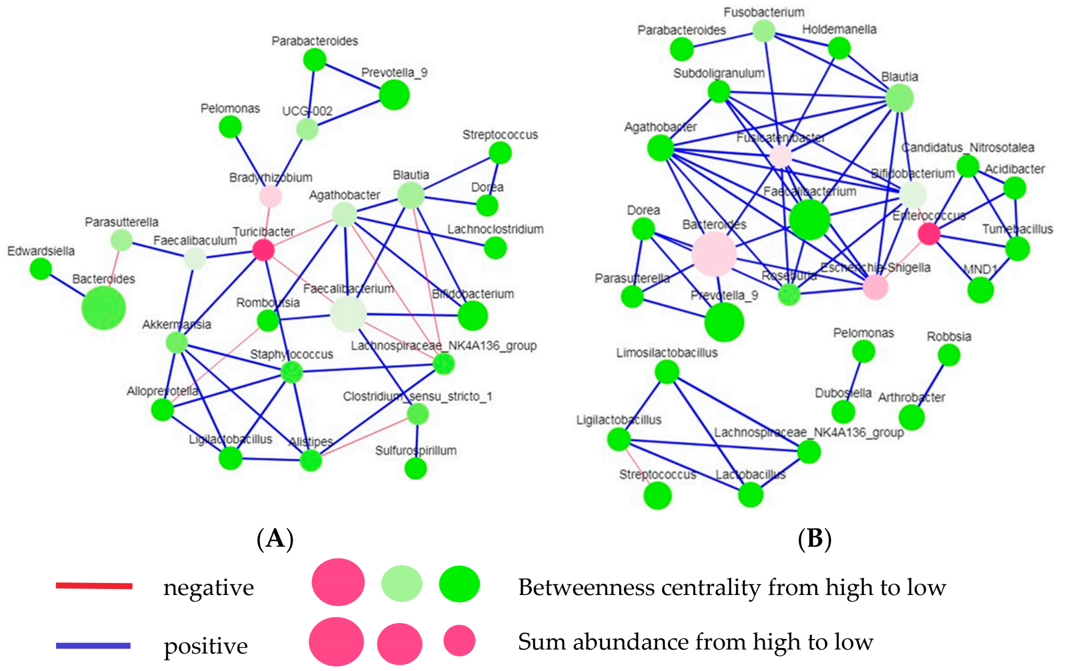

Figure S5. Co-occurrence network of bacteria in leaf of Tartary buckwheat planted in non-sterilized (**A**) and sterilized soil (**B**).

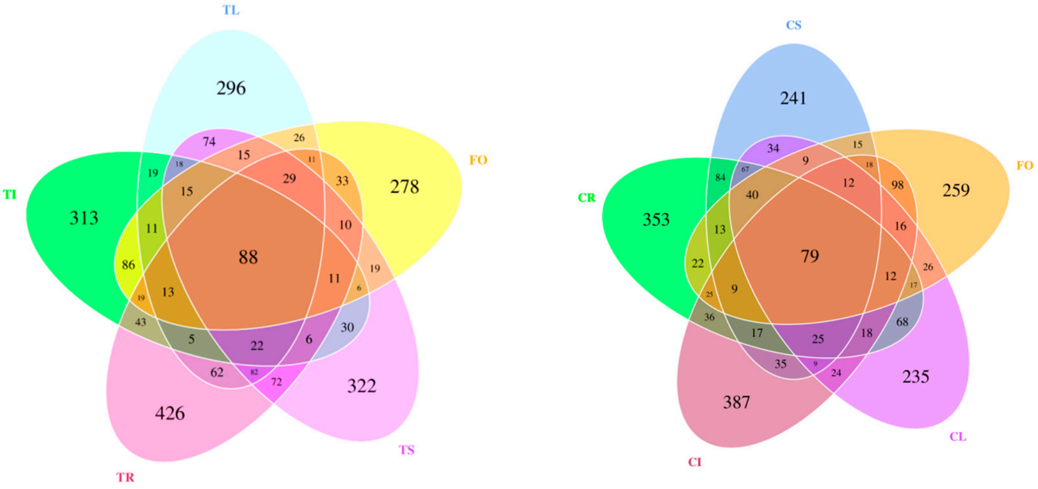

(**A**) (**B**)

Figure S6. Venn analysis of endophytic bacteria in different tissues of Tartary buckwheat in sterilized (A) and non-sterile soil (B). FO: The seeds of Tartary buckwheat germinated on MS medium for 3 days; The seeds harvested from Tartary buckwheat planted in sterilized (TI) and non-sterilized soil (CI); The leaf planted in sterilized (TL) and non-sterilized soil (CL); The stem planted in sterilized (TS) and non-sterilized soil (CS); The root planted in sterilized (TR) and non-sterilized soil (CR).

Table S1. Characteristics of effective reads and OTUs from samples of endophytic bacteria with Tartary buckwheat.

Sample	Sample origin	Number of Sample	Raw reads	Clean reads	Base(nt)	Avglen(nt)	Effective%	Total number of OTU
TL	Leaf of buckwheat planted on sterilized soil	15	78,601	74,393	18,798,822	252	94.65%	786
TS	Stem of buckwheat planted on sterilized soil	15	83,149	80,807	20,398,205	252	97.21%	819
TR	Root of buckwheat planted on sterilized soil	15	80,545	76,297	19,262,975	252	94.81%	932
CL	Leaf of buckwheat planted on non-sterilized soil	15	84,154	80,248	20,270,548	252	95.41%	691
CS	Stem of buckwheat planted on non-sterilized soil	15	80,839	79,167	20,058,295	253	97.93%	707
CR	Root of buckwheat planted on non-sterilized soil	15	82,283	77,796	19,686,047	253	94.60%	885
FO	Tartary buckwheat parent seeds (germinated on MS medium for three days)	15	82,915	80,120	20,258,535	252	96.64%	670
TI	Tartary buckwheat progeny seeds (germinated on MS medium for three days) from sterilized soil	15	82,558	80,108	20,272,455	253	97.05%	705
CI	Tartary buckwheat progeny seeds (germinated on MS medium for three days) from non-sterilized soil	15	82,328	80,116	20,270,095	253	97.34%	820

Table S2. The Alpha diversity of bacterial community in seeding and different tissues of Tartary buckwheat.

Index	Shannon	Simpson	Chao1	ACE	Observed species
FO	5.75±0.99 b [1]	0.94±0.13 b[1]	239.13±71.79 b[1]	276.54±92.97 b[1]	83±15.48 b[1]
TI	5.75±0.78 b	0.96±0.03ab	225.78±114.14b	259.42±126.14b	83±23.06b
CI	6.06±0.73ab	0.96±0.05ab	274.24±168.25ab	313.04±221.01ab	92±18.26ab
TL	6.24±0.44a	0.98±0.01ab	321.71±93.87ab	361.51±88.79ab	97±15.05ab
TR	6.22±0.63ab	0.97±0.04ab	369.91±115.82a	386.417±88.01a	100±14.69a
TS	6.46±0.26a	0.99±0.00a	315.58±127.43ab	340.77±135.07ab	103±11.22a
CL	6.13±0.40ab	0.98±0.01ab	278.85±132.95ab	323.86±141.31ab	92±14.04a
CR	6.34±0.46a	0.98±0.01ab	337.29±127.08a	401.98±162.21a	101±15.84a
CS	6.33±0.18a	0.98±0.01ab	240.75±65.36b	273.48±74.85b	96±6.46a

[1] Means ± SD followed with the same letters within each column were not significantly different ($p < 0.05$).
